# Factors associated with pediatric febrile illnesses in 27 countries of Sub-Saharan Africa

**DOI:** 10.1186/s12879-023-08350-5

**Published:** 2023-06-12

**Authors:** Paddy Ssentongo, Vernon M. Chinchilli, Khush Shah, Thaddeus Harbaugh, Djibril M. Ba

**Affiliations:** 1 Department of Medicine, Penn State Milton S. Hershey Medical Center, Hershey, PA 17033 USA; 2 Department of Public Health Sciences, Penn State College of Medicine, 500 University Drive, Hershey, PA 17033 USA; 3 Department of Biology, Millersville University, Millersville, PA USA

**Keywords:** Sub-Saharan Africa, Febrile illness, Global health, Infectious diseases

## Abstract

**Background:**

Evidence on the relative importance of various factors associated with febrile illness in children and their heterogeneity across countries can inform the prevention, identification, and management of communicable diseases in resource-limited countries. The objective of the study is to assess the relative significance of factors associated with childhood febrile illness in 27 sub-Saharan African countries.

**Methods:**

This cross-sectional study of 298,327 children aged 0 to 59 months assessed the strengths of associations of 18 factors with childhood fevers, using Demographic and Health Surveys (2010-2018) from 27 sub-Saharan African countries. A total of 7 child level factors (i.e., respiratory illness, diarrhea, breastfeeding initiation; vitamin A supplements; child's age; full vaccination; sex), 5 maternal factors (maternal education; maternal unemployment; antenatal care; maternal age, and maternal marriage status) and 6 household factors (household wealth; water source; indoor pollution, stool disposal; family planning needs and rural residence) were assessed. Febrile illness was defined as the presence of fever in 2 weeks preceding the survey.

**Results:**

Among the 298,327 children aged 0 to 59 months included in the analysis, the weighted prevalence of fever was 22.65% (95% CI, 22.31%-22.91%). In the pooled sample, respiratory illness was the strongest factor associated with fever in children (adjusted odds ratio [aOR], 5.46; 95% CI, 5.26-5.67; *P <* .0001), followed by diarrhea (aOR, 2.96; 95% CI, 2.85-3.08; *P <* .0001), poorest households (aOR, 1.33; 95% CI,1.23-1.44; *P <* .0001), lack of maternal education (aOR, 1.25; 95% CI, 1.10-1.41; *P <* .0001), and delayed breastfeeding (aOR, 1.18; 95% CI, 1.14-1.22; *P <* .0001. Febrile illnesses were more prevalent in children older than >6 months compared to those 6 months and younger. Unsafe water, unsafe stool disposal, and indoor pollution were not associated with child fever in the pooled analysis but had a large country-level heterogeneity.

**Conclusions:**

Major causes of fevers in sub-Saharan Africa could be attributed to respiratory infections and possibly viral infections, which should not be treated by antimalarial drugs or antibiotics. Point-of-care diagnostics are needed to identify the pathogenic causes of respiratory infections to guide the clinical management of fevers in limited-resource countries.

## Background

Febrile illnesses remain a major public health problem in Sub-Saharan Africa [1]. Fever is the leading cause of hospital admissions in children under five years [2]. Fever in young children usually indicates an underlying infection, but identifying the cause can pose a diagnostic challenge, particularly in low- and middle-income countries (LMICs), and malaria is more often the default diagnosis [3]. By the age of five, children from LMICs suffer an average of 40 or more episodes of fever, which healthcare providers tend to treat with antibiotics or antimalarial drugs [4]. Given that a regimen of two antibiotic prescriptions per year is considered excessive in many high-income countries [5], these estimates of cumulative pediatric exposure to antibiotics in developing countries are alarming.

As the burden of malaria continues to decrease across Africa [6] and the number of presentations for non-malarial fevers starts to increase, health care workers are faced with the challenge of managing febrile illness in children, particularly in the face of few or no diagnostic tools [3].

Immediate actions are needed to meet Sustainable Development Goal 3, i.e., end the epidemics of acquired immunodeficiency syndrome, tuberculosis, malaria, and neglected tropical diseases and combat hepatitis, water-borne, and other communicable diseases by 2030, which in turn can contribute to other targets associated with child survival, educational achievements, and overall well-being [7].

Evidence regarding the relative strengths of factors associated with childhood fever and their variation across countries is critical for understanding key factors associated with febrile illness in children. Using the most recent data from the Demographic and Health Survey (DHS), we selected a comprehensive set of factors associated with febrile illness in children. We conducted pooled analyses to assess their relative significance in 27 sub-Saharan African countries. In addition to pooled analyses, we present country-specific findings to inform each country's core intervention components to reduce childhood febrile illness.

## Methods

### Data source

We obtained the most recent data from sub-Saharan Africa from DHSs conducted between 2010-2018. The surveys are implemented approximately every 5 years by the DHS Program, with funding from the US Agency for International Development. These surveys collect nationally representative data on health information on children, their parents, and households using a multistage, stratified sampling design. The first stage involves the division of each country into geographic areas. Within these subnational regions, populations are stratified by urban or rural areas. These primary sampling units or clusters are selected with probability proportional to the contribution of that cluster's population to the total population. All households within the cluster are listed in the second sampling stage, and an average of 25 houses are randomly selected for an interview by equal-probability systematic sampling.

Survey data are available to researchers from the DHS website, conditional on registration and submission of a short research abstract. We followed the Strengthening the Reporting of Observational Studies in Epidemiology (STROBE) reporting guidelines and statement [8].

### Study population and sampling size

A total of 27 sub-Saharan African countries had collected data on the presence of fever within two weeks preceding the survey date and the factors of interest. The eligibility criteria for our analytic sample were as follows: children (1) who were aged 0 to 59 months and alive at the time of the survey, and (2) with data on the status of the fever in the preceding 2 weeks of the survey. We identified 298,327 children from 27 sub-Saharan African countries in the final analytic sample for our analysis.

### Outcomes

Fever measurement was binary (yes vs. no), indicating whether a child under the age of five had exhibited fever at any times in the 2 weeks preceding the survey. We follow the DHS convention and refer to the "prevalence" of fever as the proportion of children exhibiting symptoms during this 2-week window. The child morbidity module of the DHS asks female caregivers (ages 15–49 years) of all children younger than 5 years to report on any episodes of a fever that occurred within the 2 weeks preceding the survey.

### Exposures

We selected 18 factors for our primary analysis. We classified these 18 factors associated with febrile illness either child-level factors, maternal factors, or household factors. Seven child-level factors were identified, including, i.e., respiratory illness (defined as cough in the last two weeks and/ or short, rapid breathing which was chest-related and/or difficult breathing which was chest-related), diarrhea (defined as whether the child had diarrhea in the past 2 weeks), early breastfeeding initiation within 1 hour of birth; vitamin A supplements; child's age; full vaccination status; sex). Fully vaccinated status was defined if the child received 1 dose of Bacillus Calmette–Guerin vaccination against tuberculosis; 3 doses of diphtheria, pertussis, and tetanus vaccine; ≥ 3 doses of polio vaccine; and 1 dose of measles vaccine. Child’s age was originally reported in months and was further categorized as follow: 0-6 months, 7-24 months, and 25-59 months. Five maternal factors (maternal education; maternal unemployment; antenatal care; maternal age, and maternal marital status) and six household factors (household wealth; water source; indoor pollution, stool disposal; family planning needs and rural residence) were included. Wealth index quintiles were determined using a concept of principal component analysis of household assets (i.e., household’s ownership of several items such as car, bike, and other wealth-related characteristics). More detailed information about wealth index quintiles has been described elsewhere [9]. Sociodemographic-economic factors were assessed through self-reported questionnaires administered to all women ages 15- 49 years during the survey. Number of antenatal care visits was grouped into 2 categories: < 4 visits, and 4 or more visits.

### Statistical analysis

Consistent with the DHS guideline for analyzing the DHS data and to ensure that the estimates were nationally representative, all analyses were performed using appropriate sampling weights, clustering, and stratification to account for the complex sampling design [10]. The weighted prevalence of fever in the last two weeks was generated using SAS surveyfreq procedure (SAS Institute). The multivariable logistic regression model using the SAS surveylogistic procedure (SAS institute) was performed to determine factors associated with febrile illness adjusting for child age, child sex, the country of residence, age of mother, the number of antenatal care visits, mother educational status, marital status, wealth index status, place of residence (urban/rural), employment status, unsafe stools disposal, unsafe water, indoor pollution, diarrhea, breastfeeding within 1 hour of birth, fully vaccinated status, number of children in a household, receipt of vitamin A supplementation, and the presence of cough in 2 weeks preceding the survey. Next to better understand between-country differences, stratified analysis by country was conducted to examine the prevalence of each factor associated with fever. Descriptive statistics are presented as the weighted prevalence of fever in the last weeks. The multivariable logistic regression results were presented as adjusted odds ratio (OR) and the 95% confidence intervals (95% CIs). Data were analyzed in SAS software (version 9.4-SAS Institute) at a two-tailed alpha level of 0.05 and R statistical software version 3.4.3 (R Foundation for Statistical Computing, Vienna, Austria).

## Results

**Fig. 1:**
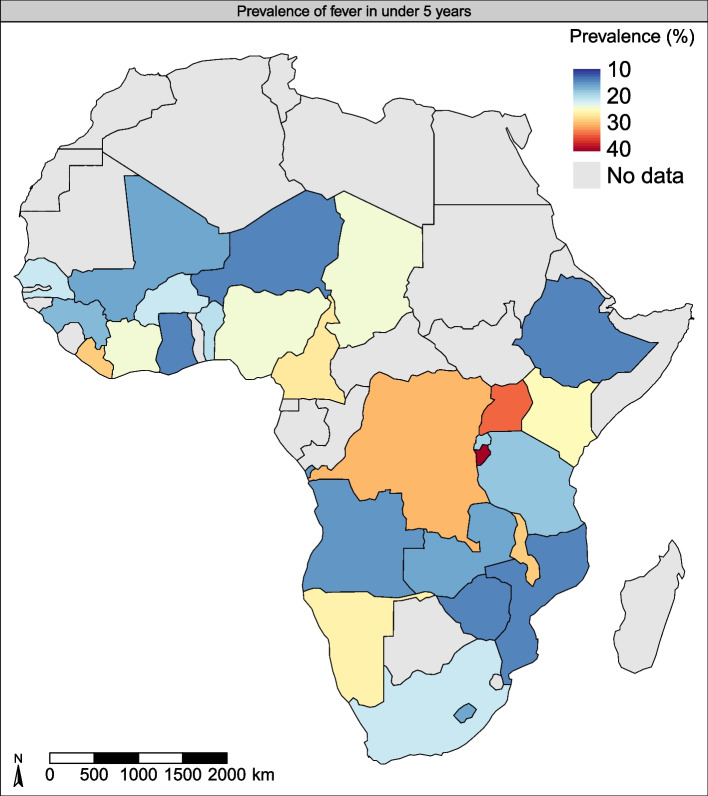
Map of Africa displaying g the prevalence of febrile illness in children < 5 years. The map was created by the author and were produced with the R software for statistical computing version 3.6.3. The world shapefile was retrieved from the spData package (version 0.3.0, https://CRAN.R-project.org/package=spData) and the maps created with the tmap package (version 3.2.2, https://CRAN.R-project.org/package=tmap)

A total of 298,327 children aged 0 to 59 months from 27 sub-Saharan African countries were included in the analysis (Fig. 1). Of these, 150,012 (50.3%) were boys, and 209,554 (70.2%) lived in rural regions (Table 1). Overall, 22.65% (95% CI, 22.31%-22.91%) of children had febrile illness two weeks preceding the survey. The prevalence of febrile illness varied widely among countries, from 13.6% (95%CI, 12.5%-14.7%) in Mozambique to 39.6% (95%CI, 37.8%-41.4%) in Burundi (Fig. 1). Overall, the burden of febrile illness was higher in older children than the younger ones, <7 months vs. 7-24 months vs. 25-59 months, 17.5% [95%CI, 17.0%-18.0%] vs. 28.5% [95%CI, 28.1%-29.0% vs. 20.6% [95%CI, 20.2%-21% ] living in rural residencies than urban regions, 23.7% [95%CI, 23.3%-24.2%] vs. 20.1% [95%CI, 19.6%-20.6%]), poorer households (fever among children with from household with lowest vs. highest wealth quintile, 24.8% [95%CI, 24.2%-25.4%] vs. 19.2% [95%CI, 18.6%-19.8%]), and those with mothers who were less educated (for example, fever prevalence in children whose mothers had no schooling vs. college education, 21.4% [95%CI, 20.9%-21.8%] vs. 17.4% [95%CI, 16.3%-18.5%]).

**Table 1:** Characteristics of the study participants

**Variable (*N*=298 327)**	**Mean ± SD or n (%)**
**Child factors**	
Age, month	29±17
Male sex	150,012 (50)
Cough in 2-weeks preceding the survey	66,023 (23)
Short rapid breaths (suspected pneumonia)†	28,882 (15)
Fully vaccinated	97,112 (42)
Had Vitamin A supplementation	139,604 (61)
Had diarrhea 2 weeks preceding the survey	47,384 (16)
Early initiation of breastfeeding (within 1 hour of birth)	161,006 (57)
**Household factors**	
Wealth index quintiles	
Lowest	73,194 (23)
Second	64,571 (22)
Middle	59,760 (20)
Fourth	53,877 (19)
Highest	46,925 (17)
Rural residence	209,554 (70)
Greater than 5 children in a household	88,684 (29)
Unsafe disposal of stool	162,751 (56)
Unsafe water source	92,844 (31)
Indoor cooking	266,945 (90)
**Maternal Factors**	
Maternal education	
No education	122,967 (39)
Elementary school	102,741 (34)
High school	63,511 (21)
Greater than high school	9083 (3)
Maternal Age Group	
15- 29	166,955 (56)
30-39	105,389 (35)
40-49	25,983 (9)
Marital Status	
Married	261,799 (88)
Not married	36,528 (12)
Unemployment status	
Yes	108,888 (37)
No	178,700 (63)
Attended > 4 antenatal care visits	97,586 (46)

### Pooled analyses

**Fig. 2:**
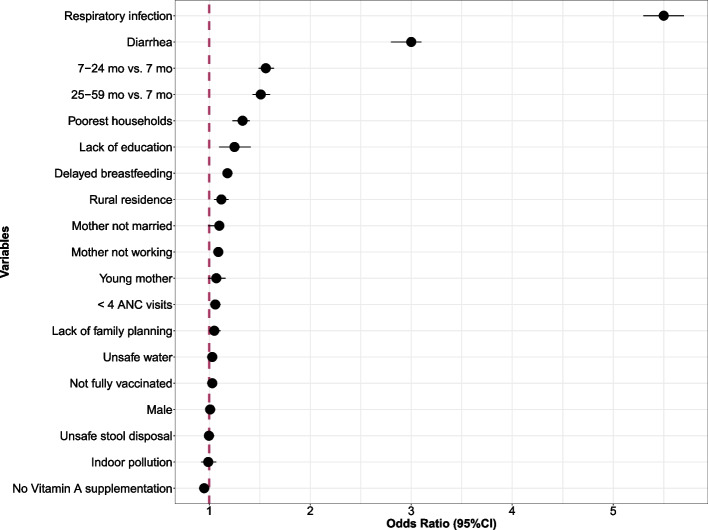
Relative Ranking of 18 Factors Associated with Febrile Illness From Multivariable Adjusted Model. Adjusted odd ratios (aOR) and their 95% confidence intervals of fever risk. The dashed vertical line in the forest plots represents the null estimate. Odds ratio greater than one indicates higher risk of fever

The full regression results from pooled analyses are presented in Table 2 and Fig. 2. In the adjusted model, respiratory illness was the strongest factor associated with fevers in children (adjusted odds ratio [aOR], 5.46; 95%CI, 5.26-5.67; *P <* .0001), followed by diarrhea (aOR, 2.96.0; 95%CI, 2.85-3.08; *P <* .0001), poorest household wealth index (aOR, 1.33; 95%CI,1.23-1.44; *P <* .0001), lack of maternal education (aOR, 1.25; 95%CI, 1.10-1.41; *P <* .0001), delayed breastfeeding (aOR, 1.18; 95%CI, 1.14-1.22; *P <* .0001 and maternal unemployment aOR, 1.09; 95%CI, 1.05-1.13; *P <* .0001), and rural residence (aOR, 1.08; 95%CI, 1.03-1.14; *P =*0.004). Unsafe water source, unsafe stool disposal, indoor pollution, full vaccination; child's sex, antenatal care; family planning needs; maternal age, and maternal marital status were not associated with febrile illnesses in the pooled analysis. Unexpectedly, lack of Vitamin A supplementation was protective against febrile illness. However, the magnitude of this association was small (aOR, 0.93; 95%CI, 0.90-0.97; *P =*0.0005).

**Table 2:** Adjusted Odds ratios for the association of child, household and maternal factors and febrile illness

**Variable**	**Odds ratio and 95% confidence interval**		
**Child factors**	**OR**	**Lower boundary**	**Upper boundary**
Age (7- 24 mo) vs <7 mo	1.54	1.46	1.63
Age 25-59 mo) vs < 7 mo	1.50	1.41	1.59
Male child	1.009	0.97	1.05
Not fully vaccinated	0.997	0.95	1.04
Delayed breastfeeding	1.19	1.14	1.24
No Vitamin A Supplement	0.95	0.90	0.99
Respiratory infection	4.27	4.04	4.51
Diarrhea	2.55	2.44	2.66
**Household factors**			
Poorest household wealth index	1.46	1.33	1.59
Rural residence	1.12	1.05	1.19
Indoor Pollution	1.10	0.93	1.1
Unsafe water	1.01	0.96	1.06
Unsafe stool disposal	0.997	0.95	1.05
**Maternal Factors**			
Maternal Unemployment	1.09	1.04	1.13
< 4 ANC visits	1.06	1.02	1.11
Lack of maternal education	1.34	1.18	1.53
Lack of family planning	1.05	1.001	1.11
Young Mother	1.07	0.99	1.16
Mother not Married	1.10	0.99	1.13

### Country-specific analyses

**Fig. 3:**
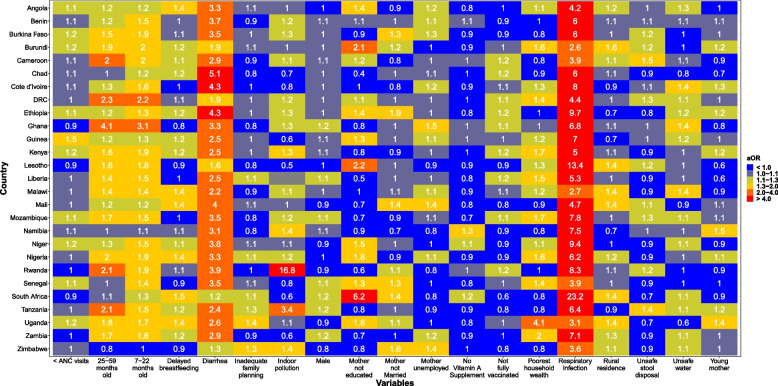
Country-Specific Odds Ratios for 18 Factors Associated With Febrile illness From Multivariable Adjusted Models

Respiratory illness had the strongest association with fevers, with ORs above 2 for all 27 countries (Fig. 3). The magnitudes of ORs for respiratory illness ranged from 2.6 (95%CI, 2.3-3.0) in Burundi to 23.7 (95%CI, 15.1-35.3) in South Africa. Respiratory illness was followed by diarrhea, which was consistently associated with fevers in most countries with ORs above 1.5 except for Lesotho, South Africa, and Zimbabwe. Although indoor pollution was not associated with febrile illness in the pooled analysis, the magnitude of association in Rwanda and Tanzania was substantial. In Uganda, poverty was the strongest factor associated with febrile illness (OR, 4.1; 95%CI, 2.9-5.7; *P =*0.0005). The magnitudes of ORs for other factors were very heterogeneous (Fig. 3).

## Discussion

In this pooled analysis of nearly 300,000 children from 27 sub-Saharan African countries, respiratory infections, diarrhea, poor household socioeconomic conditions (household wealth and maternal education) and delayed initiation of breastfeeding were the leading factors associated with childhood fevers. The relative magnitude of other factors, such as unsafe stool disposal and rural residence, showed considerable heterogeneity among countries.

Our pooled estimates of respiratory infection being the major factor associated with fever in children in sub-Saharan African countries is comparable with previous studies. A study conducted in Tanzania, a sub-Sub-Saharan African country, analyzed an extensive set of clinical, laboratory, and radiologic data in 1005 children with a fever found more than half presented with an acute respiratory infection. In 81 of these children, a virus was identified [11]. The study indicated that common childhood viral diseases are frequent in Africa even in the absence of an outbreak. Furthermore, a relatively high number of febrile children with non-malaria infections has been reported in a West African study [12]. In Africa, it has been estimated that more than 80% of children attending public clinics with fever in some countries probably don't have malaria [13]. Furthermore, a recent review stressed that among ambulatory patients, self-limiting arboviral infections and viral upper respiration infections are common, occurring in up to 60% of children attending health centers [1]. In the ambulatory patients (such as those included in this study), bacteriemia (such as *Salmonella enterica* serovar Typhi) and zoonotic bacterial infections and HIV-related opportunistic infections can occur; however they are less common than in hospitalized children with severe disease [14, 15].

Our findings have a critical implication for fever management strategies in sub-Saharan Africa. First, in the absence of severe clinical presentation such as suggestive of meningitis and bacterial pneumonia, fever should not always be treated with antibiotics [16]. This is especially important when other laboratory diagnostics are unavailable, which is true for limited-resource countries. In such an environment, detailed clinical information with or without laboratory investigation should be the cornerstone in assessing fevers in children. Respiratory infection was the most common factor associated with fever in sub-Saharan as a whole and the specific countries. A recent study of children with fever in sub-Saharan Africa indicated that less than 13% of the children with acute respiratory infection required treatment with antibiotics [11], and yet in the same setting, antibiotics are prescribed to 81% of children diagnosed with respiratory illness [4]. Second, when available, the new molecular diagnostic methods that are highly sensitive in identifying pathogens—whether colonization or causing disease—should be used as they have an added advantage of identifying putative pathogens with previously unrecognized pathogenic potential to reveal the complexity of infectious diseases in sub-Saharan Africa [17]. Our observed positive association of delayed breastfeeding initiation after birth and fever suggested that early breastfeeding initiation may play a significant role in preventing fever in low-resource areas such as sub-Saharan Africa. This could be explained by the mother’s breast milk containing antibodies and certain anti-inflammatory substances that could potentially reduce the risk of fever and keep the child hydrated. Such findings may have significant public health and clinical implications in preventing fever in these low-resource countries.

Our analysis had some limitations. First, we assumed that the 14-d period prevalence for self-reported fever from national surveys was representative of prevalence across the year. This assumption may not be valid where the causes of fever are strongly seasonal. Second, the assembled national survey data on fever prevalence rates are limited by spatial and temporal resolution. Third, we pooled data from many African countries which have varying sociodemographic, meteorological, and environmental heterogeneity. Such pooling could have introduced spurious finding. As such, our analysis should be used for hypothesis generating and country-specific longitudinal studies are warranted to elucidate the causative microbial agents of febrile illness. Fourth, febrile illness was subjective and therefore would have inflated its prevalence, however this is likely a non-differential misclassification of the outcome which generally bias the risk estimates toward the null. Lastly, the cross-sectional nature of the survey does not allow for determining causality.

## Conclusions

In this study, respiratory illness, diarrhea, and socioeconomic conditions were the strongest factors associated with fevers in children of sub-Saharan Africa. Major causes of fevers in sub-Saharan Africa may mostly be respiratory infections and possibly viral—that should not be treated by antimalarial or antibiotics. Point of care diagnostics are needed to identify the pathogenic causes of respiratory infections to guide the clinical management of fevers in limited-resource countries.

## Data Availability

The analyzed dataset is freely available from: https://dhsprogram.com/data/available-datasets.cfm. R code and data to reproduce the results in the present manuscript are archived at https://github.com/ssentongojeddy/Fevers_in_SubSaharan_Africa.

## Abbreviations

SSA: Sub-Saharan Africa
LMICs: Low- and middle-income countries
DHS: Demographic and Health Surveys
STROBE: Strengthening the Reporting of Observational Studies in Epidemiology
IRB: Institutional Review Board
aOR: Adjusted Odds Ratios
CI: Confidence Interval
HH: Household
ANC: Antenatal Care
